# Large-scale analysis of *FMR1* CGG repeat length and risk of premature ovarian insufficiency in over 92 000 women

**DOI:** 10.1093/humrep/deag061

**Published:** 2026-04-19

**Authors:** Emily J Morbey, Felix R Day, Daniel J Wright, Jack R A Murzynowski, Sinead M McGlacken-Byrne, Anna Murray, Ken K Ong, John R B Perry

**Affiliations:** IMS Epidemiology, Institute of Metabolic Science, University of Cambridge School of Clinical Medicine, Cambridge, UK; IMS Epidemiology, Institute of Metabolic Science, University of Cambridge School of Clinical Medicine, Cambridge, UK; IMS Epidemiology, Institute of Metabolic Science, University of Cambridge School of Clinical Medicine, Cambridge, UK; IMS Epidemiology, Institute of Metabolic Science, University of Cambridge School of Clinical Medicine, Cambridge, UK; Metabolic Research Laboratory, Institute of Metabolic Science, University of Cambridge School of Clinical Medicine, Cambridge, UK; University of Exeter Medical School, University of Exeter, Medical School Building, St Luke’s Campus, Exeter, UK; IMS Epidemiology, Institute of Metabolic Science, University of Cambridge School of Clinical Medicine, Cambridge, UK; Department of Paediatrics, University of Cambridge, Cambridge, UK; IMS Epidemiology, Institute of Metabolic Science, University of Cambridge School of Clinical Medicine, Cambridge, UK; Metabolic Research Laboratory, Institute of Metabolic Science, University of Cambridge School of Clinical Medicine, Cambridge, UK

**Keywords:** *FMR1*, premature ovarian insufficiency, whole genome sequencing, menopause, fragile X-associated primary ovarian insufficiency

## Abstract

**STUDY QUESTION:**

Does *FMR1* repeat length confer clinically meaningful predictive value for premature ovarian insufficiency (POI)?

**SUMMARY ANSWER:**

*FMR1* repeat length increases POI risk from ∼36 repeats onward but has limited diagnostic utility compared with a polygenic score for menopause timing.

**WHAT IS KNOWN ALREADY:**

*FMR1* premutation carriers (≥55 repeats) are reported to have high risk of Fragile-X Associated Primary Ovarian Insufficiency (FXPOI), but prior studies were small and highly ascertained.

**STUDY DESIGN, SIZE, DURATION:**

Cross-sectional analysis of ∼92 000 women from the UK Biobank with genetic and health data.

**PARTICIPANTS/MATERIALS, SETTING, METHODS:**

Female UK Biobank participants were genotyped for *FMR1* repeat length. Associations with self-reported POI, age at menopause, and other reproductive phenotypes were analysed in women. *FMR1* protein levels were measured, and genome-wide analyses were conducted to identify potential genetic modifiers.

**MAIN RESULTS AND THE ROLE OF CHANCE:**

Of 518 female premutation carriers with available age at natural menopause, only 6.9% reported POI. Elevated POI risk was observed starting at 36 repeats, increasing continuously with repeat length, but no threshold showed strong predictive power (maximum AUC 0.60 vs AUC 0.64 for polygenic score). No association was found between repeat length and *FMR1* protein levels, consistent with an RNA gain-of-function toxicity mechanism. *RAD52* was identified as a potential genetic modifier.

**LARGE SCALE DATA:**

UK Biobank resource (https://www.ukbiobank.ac.uk).

**LIMITATIONS, REASONS FOR CAUTION:**

POI was self-reported rather than clinically confirmed. Analyses could not assess AGG interruptions, mosaicism, or X-inactivation. Genetic modifiers require replication. Findings are limited to a single population dataset.

**WIDER IMPLICATIONS OF THE FINDINGS:**

These results challenge the utility of the FXPOI disease category, suggest limited diagnostic value of clinical *FMR1* premutation testing for POI, and highlight alternative mechanisms and potential modifiers such as *RAD52*.

**STUDY FUNDING/COMPETING INTEREST(S):**

This work was conducted using the UK Biobank resource (application 9905). This work was funded by the Medical Research Council (unit programs: MC_UU_12015/2, MC_UU_00006/2) and Wellcome (Discovery award 302536/Z/23/Z). The sponsors had no role in the study design, collection, analysis, or interpretation of the data, the writing of the manuscript or the decision to submit it for publication. J.R.B.P. and A.M. have engaged in paid consultancy for Ovartix Ltd.

**TRIAL REGISTRATION NUMBER:**

N/A.

## Introduction

Premature ovarian insufficiency (POI) is a heterogeneous disorder characterized by a loss of ovarian function in women younger than 40. It has a prevalence of around 3.5% in the general population (Li *et al.*, 2023). POI results in premature loss of ovarian endocrine function and is a leading cause of infertility, and has other comorbidities including depression, reduced bone mineral density, and increased risk of cardiovascular disease (Rudnicka *et al.*, 2018; Tassone *et al.*, 2023).

POI has a highly heritable component, with the majority of cases likely explained by a complex mixture of common genetic effects and environmental risk factors such as smoking (Ruth *et al.*, 2016b, 2021; Shekari *et al.*, 2023). There are also a number of genes implicated in monogenic forms of POI (Shekari *et al.*, 2023), largely implicating DNA damage response genes such as *MCM8*, and *STAG3*, where mutations impair the ability to complete meiosis (Le Quesne Stabej *et al.*, 2016) and effectively process double-strand breaks (Desai *et al.*, 2017). Repeat expansion of a CGG trinucleotide repeat within the 5′ untranslated region of *FMR1* is the most widely reported cause of POI. A typical repeat range of 6–44 (Rossetti *et al.*, 2017), with a full mutation described as having 200 or more repeats, resulting in hypermethylation and loss of expression of the *FMR1* gene and protein levels, and subsequently the severe neurodevelopmental condition fragile X syndrome (Barasoain *et al.*, 2016; Noto *et al.*, 2016; Rossetti *et al.*, 2017). A repeat of 55–200 bases is classified as a premutation and in this range is considered unstable, such that there is a risk of repeat expansion to a full mutation in the next generation (Noto *et al.*, 2016; Tabolacci *et al.*, 2022). The premutation results in increased levels mRNA transcript and lower levels of FMRP protein product (Tassone *et al.*, 2000; Kenneson *et al.*, 2001), although uncertainty remains around the magnitude of effect on FMRP levels (Peprah *et al.*, 2010). Given that the premutation range of 55–200 repeats is defined based on repeat instability, there remains scope to investigate the true threshold for this RNA-mediated gain-of-function effect.

It is estimated that 20% of women with *FMR1* premutations develop POI (Sherman *et al.*, 2005), which is termed Fragile X-Associated POI (FXPOI) (Barasoain *et al.*, 2016), *FMR1* is the only gene currently recommended for routine testing in the diagnostic setting (in addition to standard sex chromosomal analysis), and premutations in the 55–200 range are the focus of testing. The European Society of Human Reproduction and Embryology’s (ESHRE) guidelines on the management of POI include that *FMR1* premutation testing is recommended for all women with non-iatrogenic POI (The ESHRE Guideline Group on POI *et al.*, 2016). However, due to a lack of well-powered studies, the penetrance of repeats at different thresholds on POI remains unclear. *FMR1* testing also currently follows a ‘phenotype-first’ approach, relying on affected individuals and their family members, which often substantially overestimates the penetrance of pathogenic variants (Wright *et al.*, 2024). Therefore, the presence of this premutation may not necessarily reflect the cause of a woman’s POI (Tassone *et al.*, 2023). In addition, there is uncertainty regarding the number of repeats that are causally associated with POI; the risk associated with carrying ‘intermediate’ range alleles (45–54 repeats) remains unclear (Voorhuis *et al.*, 2014; Barasoain *et al.*, 2016).

To date, the relationship between *FMR1* repeat length and ovarian ageing has mainly been assessed in relatively small clinical studies (N∼1000). The largest population-based study to date of the impact of the *FMR1* expansion on early menopause and POI was conducted in 2135 women with natural menopause before 46 years, and 1915 controls (Murray *et al.*, 2014). However, the recent availability of large-scale population-based cohorts now allows ‘genotype-first’ estimates of penetrance for many conditions. We sought to build on existing work investigating the role of *FMR1* on POI by investigating the impact of *FMR1* in the UK Biobank. Using data from 92 557 women, we found a robust association between *FMR1* premutation status and POI, but with lower penetrance than has previously been reported. We also show modest predictive power of the *FMR1* premutation on POI, and a more continuous risk model beginning below the traditional 55-repeat cutoff. Our findings also reinforce RNA toxicity, opposed to FMRP deficiency, as the likely pathogenic mechanism.

## Materials and methods

### Population characteristics and phenotypes in the UK Biobank

This study was conducted using the UK Biobank (UKBB) Resource under approved application number 9905. UK Biobank has ethical approval from the North West Multi-centre Research Ethics Committee (MREC) and all participants provided written informed consent for use of their data and samples in health-related research. No additional ethical approval was required for this secondary data analysis in accordance with the UK Biobank’s governance framework.

Unrelated individuals of white European ancestry from UKBB, based on previously defined ancestry groups (Ruth *et al.*, 2021), with measures of *FMR1* repeat length was included in this study.

The age at natural menopause (ANM) phenotype for women was derived as outlined in Stankovic *et al.* (2024), and represents women who are deemed to have undergone natural menopause, not affected by surgical or pharmaceutical intervention. Menopausal status was determined using data across instances (field 2724) and prioritizing the latest reported data to account for changes in menopause status. Exclusions to these phenotype were carried out for the following participants; participants having undergone a hysterectomy or oophorectomy before or during the year they report undergoing menopause, women who had undergone oophorectomy and/or hysterectomy and not reported the age at which this occurred, participants reporting multiple hysterectomy and/or oophorectomy ages, which were more than 10 years apart, participants starting HRT prior to undergone menopause or those reporting HRT use with no accompanying dates, participants reporting multiple HRT start and/or end ages not in chronological order and/or more than 10 years apart, participants reporting multiple ages at menopause which were not chronologically ascending and/or were more than 10 years apart, The resulting trait was representative of ANM. POI was defined as reaching menopause before 40 years of age, and early menopause defined as reaching menopause before 45 years of age. After excluding related women, not of European ancestry, a final sample of 198 307 was available for further analyses. Of these women, 92 557 had available data on ANM.

For age at menarche (AAM), the phenotype was derived using data from field 2714 and as outlined in Kentistou *et al.* (2024). To maximize sample size, individuals with missing or implausibly early or late ‘first instance’ AAM (<8 years old or >19 years old) were imputed using data from the next available instance (Kentistou *et al.*, 2024). Menstrual cycle length was obtained from data field 2724, in which women who indicated they had not yet reached menopause were asked ‘How many days is your usual menstrual cycle?’. Users of hormonal contraceptives, as indicated in fields 2804, 41272, and field 20003 were excluded. This data field and these exclusions were also used to obtain a binary irregular periods phenotype, as women could respond ‘irregular cycle’ within this question. Number of live births was obtained from data field 2734. Age at last birth was obtained from data field 2764.

### Detection of *FMR1* repeat length in from WGS data using expansion hunter

We utilized the catalogue of short tandem repeats (STRs) identified using ExpansionHunter (EH) (Dolzhenko *et al.*, 2017, 2019, 2020) software (v5.0) with DRAGEN Bio-IT Platform (v3.7.8) on whole genome sequencing data available for ∼500 000 participants in UKBB (accessed from the UK). CGG repeat lengths at the *FMR1* locus were quantified by UKBB using ExpansionHunter and made available via the UKBB Research Analysis Platform (RAP) in Variant Call Format (VCF). We extracted *FMR1* repeat lengths from these VCF files and linked them with participant identifiers to determine the number of CGG repeats in *FMR1* in women in UKBB. As a sensitivity analysis, we evaluated the zygosity of *FMR1* repeat lengths using ExpansionHunter output by comparing repeat lengths across the two alleles. We found that ∼19% of women appeared homozygous at this locus, which was suitable given the expected homozygosity of ∼15% at this locus.

### Modelling the effect of *FMR1* repeat length on POI and ANM

All analyses were generated in the RStudio environment (v4.4) in the DNAnexus Research Analysis Platform (RAP) (accessed from the UK). *P*-values <0.05 were considered to be statistically significant.

To determine the most suitable diagnostic threshold for an *FMR1* premutation, we ran a series of logistic regressions in which the exposure was defined by varying trinucleotide repeat thresholds. Specifically, we incrementally increased the repeat length cutoff one repeat at a time and compared women above each threshold to those with a repeat length of 30 (n = 38 238). This was done using the maximum CGG repeat length observed across the two alleles. To retain statistical power, repeats higher than 75 at the maximum allele were grouped together.

To assess whether total repeat burden provided additional predictive value, we then repeated this process using the sum of the length of alleles, with a reference value of 60 (n = 15 350), to determine whether the maximum allele, or the sum of alleles was more strongly associated with POI. All models adjusted for age, sequence provider, mean read depth, and the first 20 genetic principal components (PCs) to account for population structure.

To discriminate which premutation threshold might best predict the POI phenotype, we also calculated the sensitivity, specificity, and AUC for each model using the pROC (Robin *et al.*, 2011) package in R, and compared the ROC curves for the subsection of models which showed the most significant *P*-values in the thresholding models. Based on the AUC values for each threshold and the results from the series of logistic regressions, a premutation value of 55 repeats was taken forward for further modelling as the binary premutation exposure.

### Investigating the effect of the *FMR1* STR on other phenotypes

To model the associations between the *FMR1* STR and various phenotypic outcomes, we operationalized the genetic exposure in distinct ways for female participants. First, we considered the presence of a premutation, defined by the conventional threshold of ≥55 CGG repeats. Second, we examined the length of the longer allele (the maximum CGG repeat count across the two X chromosomes). Third, we analysed the total repeat burden by summing the CGG repeat lengths of both alleles, reflecting potential additive effects.

Logistic and linear regression models were then used to assess the association between these exposures and POI, ANM, and AAM, as well as menstrual cycle characteristics and number of children ever born. We also modelled the association between maximum allele length and ANM and AAM using natural cubic spline regression with knots at 36 and 55 repeats, due to the observed non-linear association between maximum allele length and these outcomes. All models adjusted for age, sequence provider, mean read depth, and 20 PCs.

To measure the association between *FMR1* repeat length and FMRP protein levels, and the association between FMRP protein levels and age at menopause, we obtained information on FMRP levels from UKBB from the OLINK protein data field 143, coding 1070 and matched to our dataset based on participant ID. Protein levels were available for 20 062 of the 198 307 unrelated European women for whom *FMR1* repeat length were available. We used linear regressions to model the association between *FMR1* trinucleotide repeat length and FMRP levels in these 20 062 women. All models adjusted for age, sequence provider, mean read depth, and 20 PCs.

### Interaction models with genetic and environmental exposures

To investigate potential causes of incomplete penetrance of the *FMR1* premutation, we modelled the interaction between known genetic and environmental factors influencing POI and ANM. Firstly, we modelled the potential interaction of the *FMR1* premutation with the genetic risk score for ANM on POI and ANM in women. The genetic risk score for ANM was derived from 290 independent, genome-wide significant signals in Stankovic *et al.* (2024). We coded this score additively and aligned it to the menopause age-decreasing direction. We then ran separate logistic regression models with POI and ANM as the dependent variables, with the carrier status of a premutation (≥55 repeats) as the main independent variable, and the ANM decreasing polygenic risk score (PRS) as an interaction term. These models controlled for age, sequence provider, mean read depth, array batch, and 20 PCs.

To evaluate any participation bias based on menopausal age, we regressed the standard PRS for age at menopause from UKBB on sex, due to this data being available in both men and women (Thompson *et al.*, 2024).

To investigate interactions between the *FMR1* premutation and specific loci from within from the 290 signal ANM PRS, we conducted separate logistic regression models for each SNP. In each model, the SNP genotype (coded additively and aligned to the menopause decreasing direction) and *FMR1* premutation status were included along with their interaction term, to test whether the association between the premutation and POI different by SNP genotype.

Environmental exposures that we tested for an interaction with the *FMR1* repeat included smoking status, BMI, alcohol consumption, previous use of hormonal oral contraceptives, number of live births, and pelvic surgery. Smoking status was derived from field 20116 in UKBB, where participants self-reported their smoking status as either ‘Never’, ‘Previous’, or ‘Current’. Individuals who reported that they would ‘Prefer not to say’ or ‘Do not know’ were excluded from the analysis. Smoking was modelled as a categorical factor variable with three levels. BMI was derived from field 21001 in UKBB and categorized as a levelled factor variable as ‘Normal’ (18.5–24.9 kg/m^2^), ‘Overweight’ (25–29.9 kg/m^2^), and ‘Obese’ (>30kg/m^2^). Alcohol consumption was derived from UKBB field 1558 and grouped into ‘Frequently’ (‘Daily or almost daily’, ‘Three or four times a week’), ‘Sometimes’ (‘Once or twice a week’), ‘Rarely’ (‘One to three times a month’, ‘Special occasions only’), and ‘Never’. Previous use of hormonal oral contraceptives was obtained from UKBB field 2784 where participants were asked whether they had ever taken the oral contraceptive pill. Individuals who answered ‘Do not know’ and ‘Prefer not to answer’ were excluded, resulting in a binary ‘Yes’, ‘No’ variable. Number of live births was derived from UKBB field 2734 and categorized into 0, 1, 2, 3, or ≥4 births. Previous pelvic surgery was derived from the Office of Population Censuses and Surveys Classification of Interventions and Procedures, version 4 (OPCS-4) (*National Clinical Coding Standards OPCS-4 2025 [V12.0]*, 2025) codes in UKBB (field 41272). All surgeries of the upper female genital tract (chapter Q) were combined to generate a pelvic surgery exposure.

Interaction terms between *FMR1* repeat length and each environmental factor were included in the logistic regression models. These models controlled for age, sequence provider, mean read depth, and 20 PCs.

## Results

### Prevalence of *FMR1* premutation in women with early menopause and POI

There were 198 307 unrelated women in the UK Biobank with data on *FMR1* repeat length. Women were grouped by existing clinical thresholds of *FMR1* repeat length, namely ‘Normal’, ‘Intermediate’, and ‘Premutation’ to compare descriptive characteristics (Table 1).

**Table 1.** Descriptive characteristics of unrelated European women in the UK Biobank based on clinically defined premutation carrier status.

	Normal (<45)	Intermediate (45–54)	Premutation (>55)
	(95% CI)	(95% CI)	(95% CI)
**N**	191 464 (96.55%)	5818 (2.93%)	1025 (0.52%)
**Age (mean)**	56.53 (56.50–56.57)	56.43 (56.23–56.64)	56.79 (56.30–57.28)
**Proportion with POI %**	2.18 (2.09–2.28)	1.94 (1.48–2.52)	6.94 (5.06–9.47)***
**Max allele (mean)**	31.1 (31.1–31.2)	46.9 (46.7–47.0)	64.9 (64.0–65.9)
**Sum alleles (mean)**	57.5 (57.4–57.5)	75.9 (75.8–76.1)	94.1 (93.1–95.1)
**ANM (mean)**	50.1 (50.0–50.1)	50.1 (49.9–50.2)	49.0 (48.6–49.5)***
**AAM (mean)**	12.96 (12.95–12.96)	12.92 (12.88–12.96)	13.00 (12.91–13.10)
**N children (mean)**	1.80 (1.79–1.80)	1.80 (1.77–1.83)	1.81 (1.74–1.88)
**Menstrual cycle length (mean)**	27.5 (27.4–27.6)	27.5 (27.4–27.7)	27.4 (27.0–27.8)
**Lost pregnancies (mean)**	0.70 (0.70–0.71)	0.69 (0.68–0.72)	0.69 (0.63–0.74)
**BMI**	26.99 (26.98–27.00)	26.87 (26.86–26.88)	27.02 (27.01–27.03)
**Ever smoked (%)**	41.23 (41.00–41.44)	38.81 (37.56–40.08)***	42.93 (39.88–46.03)

*** *P *< 0.001 in pairwise comparisons.

The analytical sample of unrelated European women with *FMR1* repeat length and data on ANM included 92 557 women. This sample included 2035 women with POI (2.20%) and 90 522 women without POI (97.80%). A total of 9489 (10.25%) had early menopause (<45 years old) (including POI). The repeat length of *FMR1* ranged from 5 repeats to 188 repeats. A single woman with a full mutation (>200 repeats) was excluded from further analyses. Overall, 518 (0.56%) women had a *FMR1* premutation (one allele of 55 repeats or longer; Tassone *et al.*, 2023), and this was more common in women with POI (1.8%) than in controls (0.53%). Two women carried two alleles with 55 or more repeats and reported menopause ages of 47 and 49 years.

**Figure 1.**
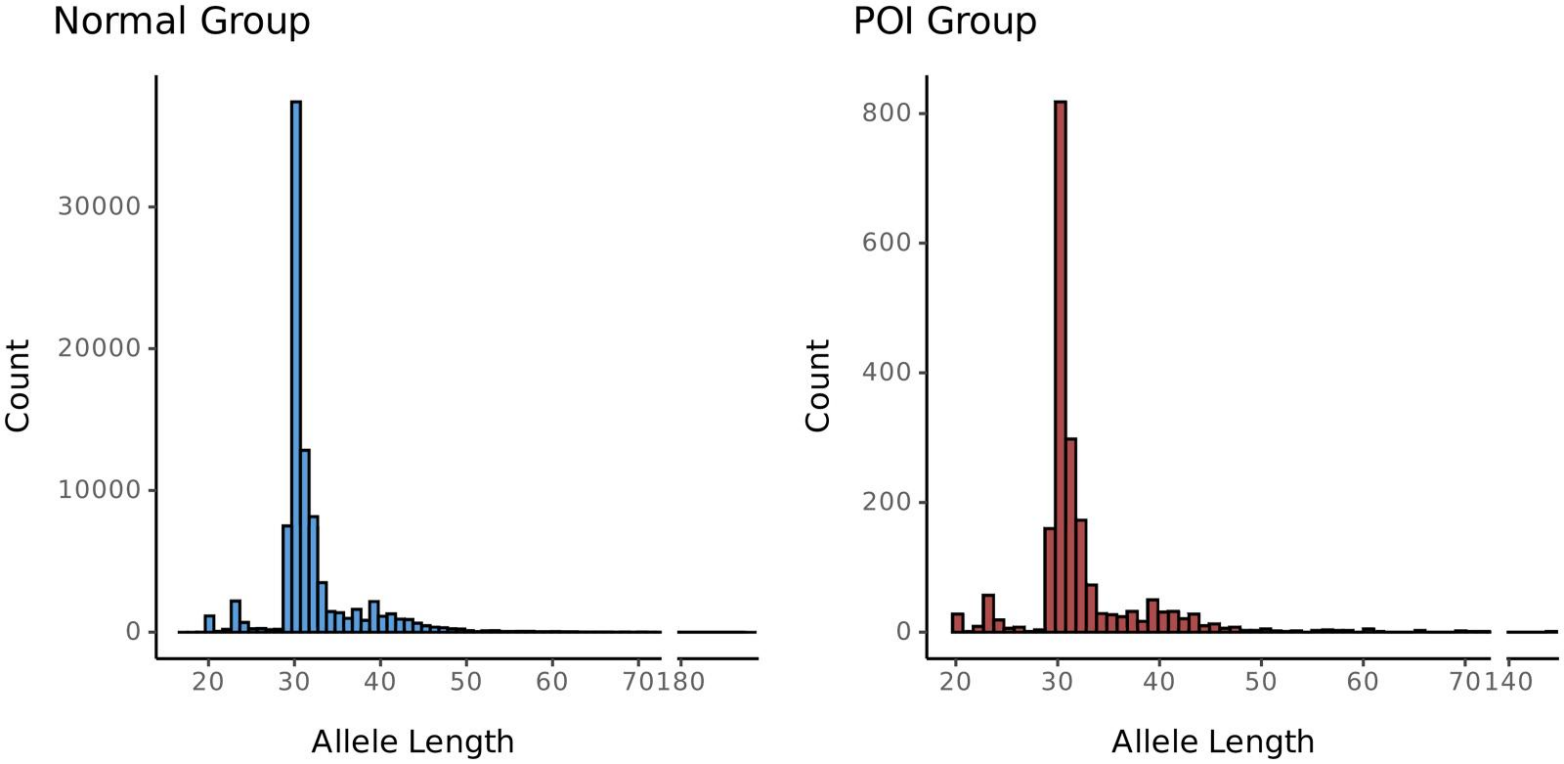
**Histograms depicting maximum allele length of the *FMR1* short tandem repeat among women without premature ovarian insufficiency (POI) and women with premature ovarian insufficiency in the UK Biobank.** Histograms show a similar distribution of CGG repeat length on the maximum allele across POI cases and controls.

The maximum allele in the overall sample showed a distribution consistent with that in clinical studies, with a mode at 30 copies, and minor peaks at 20 and 23 copies (Fu *et al.*, 1991; Peprah, 2012; Ruth *et al.*, 2016a). However, a peak was also observed at 41 copies. The maximum allele length showed a similar distribution in women with POI and those without POI (Fig. 1). Ninety-six percent of women with POI had a maximum and minimum allele within the typical range of below 44 repeats, 2.2% of women with POI had a maximum allele in the intermediate range, and only 1.8% of women with POI had a maximum allele that measured 55 repeats or longer.

### Investigating the clinical threshold of *FMR1* premutations

To evaluate the most suitable diagnostic threshold for defining an *FMR1* premutation in the context of POI, we conducted a series of logistic regression models, incrementally varying the threshold for the CGG repeat length on the maximum allele. For each model, individuals with a maximum allele length equal to or greater than the threshold, we compared to those with 30 CGG repeats, owing to this being the modal max allele length. This allowed us to assess the odds of POI at each discrete repeat length, helping to inform potential clinical cutoffs.

**Figure 2.**
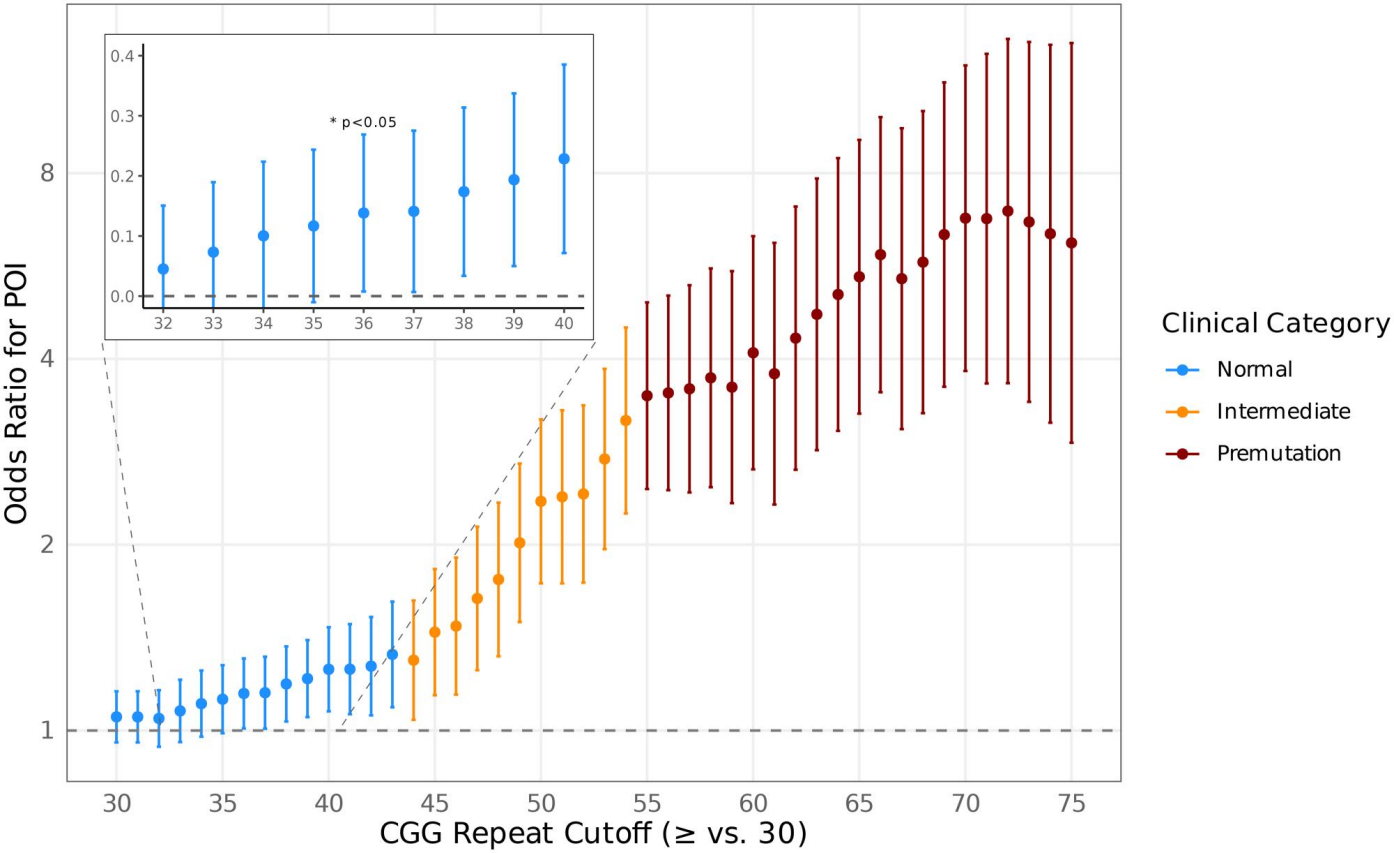
**Association between *FMR1* repeat length and risk of primary ovarian insufficiency.** Odds ratios (ORs) and 95% confidence intervals for premature ovarian insufficiency (POI) are shown for increasing CGG repeat cutoffs (≥repeat number vs 30 repeats). Estimates are stratified by clinical category: Normal (blue), Intermediate (orange), and Premutation (red). The dashed horizontal line indicates OR = 1. The inset panel magnifies the Normal range to illustrate the modest but progressive increase in POI odds, with an OR statistically significantly above 1 where *P* < 0.05.

In the thresholding model, a nominally statistically significant elevated odds ratio for POI was observed at all thresholds greater than at 36 repeats (OR = 1.14, 95% CI: 1.01–1.31, *P *= 0.04), with 2.4% of women above this threshold having POI (*n *= 324 of 13 361). The strongest statistical association occurred at the premutation 55-repeat threshold, where the odds of POI were significantly elevated (OR = 3.49, 95% CI: 2.46–4.94, *P *= 2.00 × 10^−12^; 6.9% with POI, *n *= 36 of 518). The largest effect estimate was observed at a threshold of 72 repeats, with an odds ratio of 6.94 (95% CI, 3.65–13.20, *P *= 3.29 × 10^−9^), although based on a smaller sample size (12.6% with POI, *n *= 11 of 86). A declining trend in the odds of POI was observed from thresholds of 72 repeats and higher, but it is unclear whether this reflects a biological association, or reduced statistical power at very high repeat lengths (Fig. 2, Supplementary Table S1).

**Figure 3.**
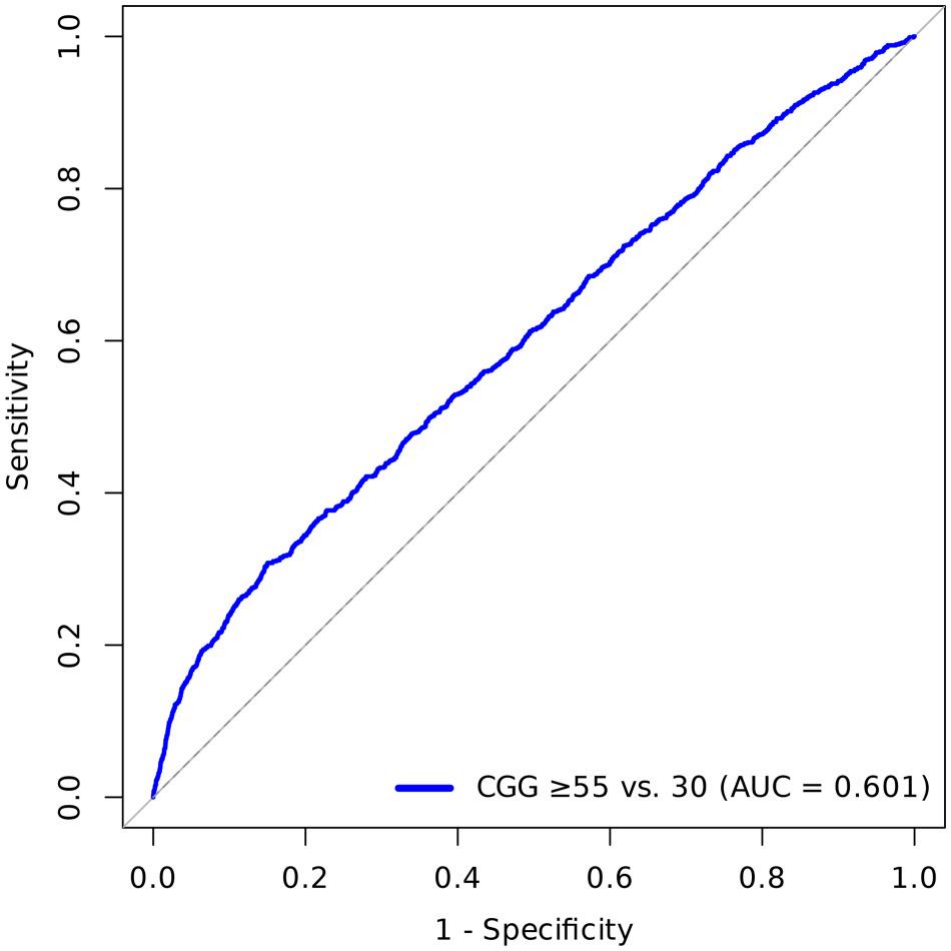
**Receiver operating characteristic curve for CGG ≥ 55 repeats in predicting primary ovarian insufficiency.** Receiver operating characteristic (ROC) curve showing sensitivity versus 1−specificity for a CGG repeat cutoff of ≥ compared with 30 repeats. The area under the curve (AUC) was 0.601, indicating modest discriminatory ability. The diagonal grey line represents no discrimination (AUC = 0.5). The AUC of 0.601 suggests limited predictive utility of this cutoff alone for identifying POI.

To further investigate which premutation threshold was the best predictor of POI, we calculated the AUC of each threshold around the current clinical threshold of 55 repeats (Hunter *et al.*, 1993; Tassanakijpanich *et al.*, 2021). The highest observed AUC was 0.601 at 55 repeats (Fig. 3), as well as at other thresholds between 50 and 58 repeats (Supplementary Table S2). This low penetrance is reflected in the fact that among those with POI in our sample, only 1.8% had a premutation, and of those with a premutation, only 6.9% had POI.

**Figure 4.**
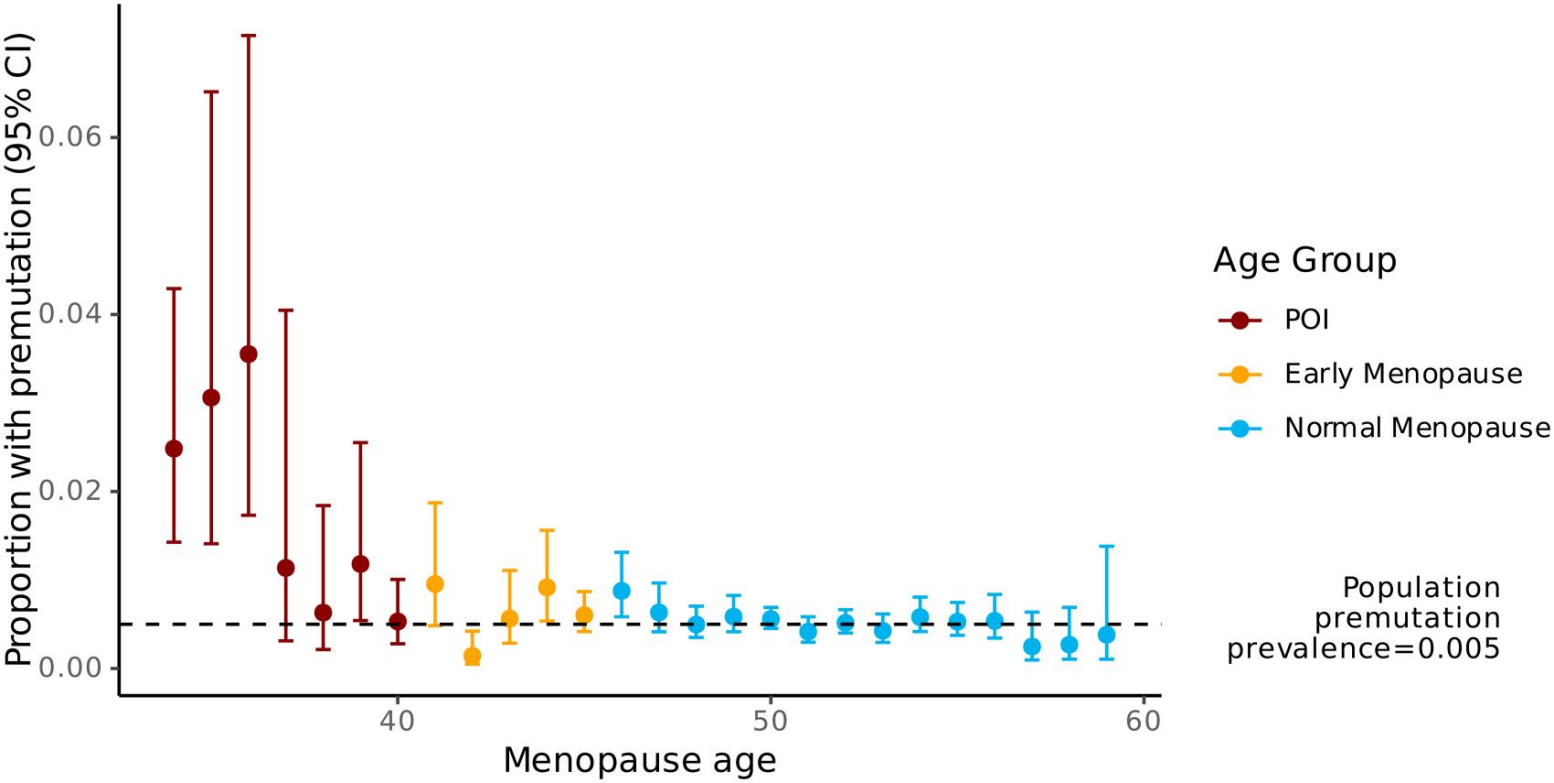
**Proportion of *FMR1* premutation carriers by menopause age.** The proportion of women with an *FMR1* premutation is shown across menopause categories, stratified as primary ovarian insufficiency (POI), early menopause, and normal menopause. Points represent estimated proportions and error bars indicate 95% confidence intervals. The dashed horizontal line represents the estimated population premutation prevalence (0.005). Premutation prevalence is highest among women with POI and declines progressively with increasing menopause age, suggesting that the premutation primarily influences risk of pathologically early menopause rather than shifting menopause age more broadly.

We next examined the prevalence of *FMR1* premutation carriers across the distribution of menopause age, to explore how carrier frequency varies with reproductive timing. The proportion of premutation carriers is markedly elevated among individuals with menopause onset before age 36 years, with those experiencing menopause before age 36 years showing prevalence estimates of around 4%, which substantially exceeds the general sample prevalence of around 0.5% (Fig. 4). This suggests that the premutation primarily influences risk of pathologically early menopause rather than shifting menopause age more broadly given we saw no protective effect at later menopausal ages.

### Modelling the association between *FMR1* repeat length and other phenotypes

We then investigated the impact of the *FMR1* repeats on other reproductive phenotypic outcomes using three distinct genetic exposures for the *FMR1* repeats; the presence of a premutation, defined by the conventional threshold of ≥55 CGG repeats, the length of the longer allele (the maximum CGG repeat count across the two X chromosomes), and the total repeat burden by summing the CGG repeat lengths of both alleles.

**Figure 5.**
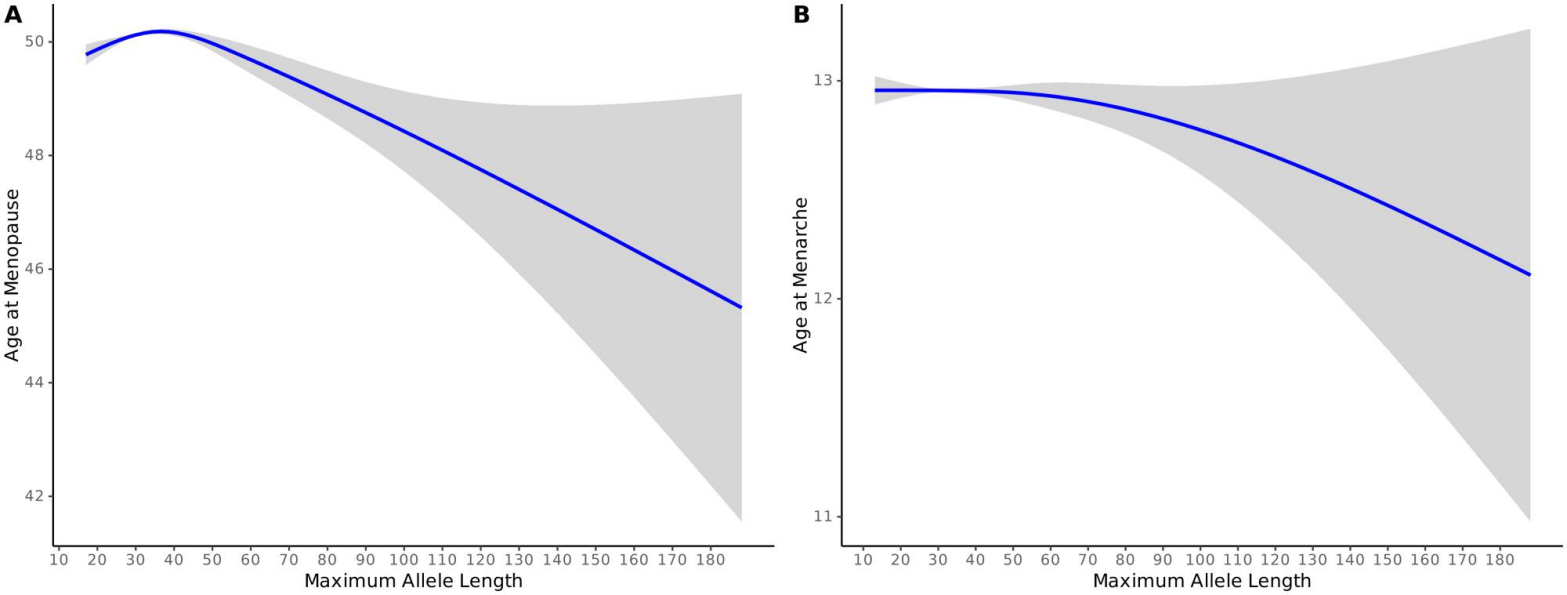
**Spline regression using natural cubic splines with knots at 36 and 55 repeats.** (**A**) Age at menopause, (**B**) Age at menarche. Knots set to reflect visual change in distribution shape at these repeat lengths. Shaded area represents 95% confidence intervals. Age at menarche does not show a significant association with CGG repeat length in spline regression, while age at menopause shows significant associations for all segments, synonymous with the increasing POI risk at longer CGG repeat lengths.

The presence of a premutation, maximum allele length, and the sum of alleles all showed significant effects on the odds of having POI (Premut: OR: 3.43, 95% CI, [2.43–4.84], *P *= 2.00 × 10^−12^, Max allele: OR = 1.01 per additional repeat, 95% CI [1.01–1.02], *P = *3.22 × 10^−4^, Allele sum: OR = 1.007 per additional repeat, 95% CI [1.001–1.012], *P = *0.014). However, when analysing continuous age at menopause only the presence of a premutation showed an association (Premut: *β*=−0.98, 95% CI [−1.36 to −0.60], *P *= 4.14 × 10^−7^, Max allele: *β*=−0.004, 95% CI [−0.009 to 0.002], *P = *0.19, Allele sum *β* = −8.612e−07, 95% CI [−0.004 to 0.004], *P = *0.9996). Neither the presence of a premutation, nor the maximum allele length were associated with AAM, but the allele sum showed a small significant association in linear regression models (Premut: *β *=  0.05, 95% CI, [−0.049 to 0.15], *P *= 0.32, Max allele: *β* = −0.0006, 95% CI [−0.002 to 0.0008], *P = *0.38, Allele sum *β* = −0.001, 95% CI [−0.002 to −0.0001], *P = *0.03). Based on these results and a visually non-linear association between allele length and ANM and AAM, non-linear models were constructed using natural cubic splines with knots at 36 and 55 repeats for these outcomes. For ANM, each of the three spline terms were significantly associated with ANM (Fig. 5, Supplementary Table S3). For AAM, none of the spline terms reached statistical significance (Supplementary Table S3).

None of the models for the *FMR1* repeats were associated with number of live births, age at last birth, number of lost pregnancies, menstrual cycle length, or irregular periods (Supplementary Table S4). None of the genetic exposures representing the *FMR1* repeats showed associations with FMRP protein levels in linear regressions (Supplementary Table S5, Supplementary Fig. S1).

### *FMR1* repeat length interaction with genetic and environmental exposures

Given the incomplete penetrance of the *FMR1* premutation, we investigated potential genetic and environmental interactions. Using the PRS derived from 290 common genome-wide significant loci for ANM, we found no significant difference in the PRS effects on ANM or POI risk between premutation carriers and non-carriers (Supplementary Table S6, Supplementary Fig. S2) Using the UK Biobank standard PRS for age at menopause, we found no association of the score with genetic sex, suggesting no participation bias related to menopause timing (Pirastu *et al.*, 2021) (*β*_Female_ = 0.003, 95% CI [−0.003 to 0.009], *P = *0.33) (Supplementary Fig. S3). SNP-level interaction testing between the *FMR1* premutation and each of the 290 loci revealed two loci, rs4972504 (*β*_interaction_ = −1.24, *P *= 2.6 × 10^−05^) and rs60378595 (*β*_interaction_ = 1.27, *P *= 9.5 × 10^−05^), with significant interactions on ANM (Supplementary Table S7). Neither of these two signals is definitively linked to the function of a nearby gene, however, rs60378595 is ∼15Kb from *RAD52*, a key mediator of homologous recombination that facilitates the repair of double-strand DNA breaks by promoting the annealing of complementary DNA strands (Nogueira *et al.*, 2019). Further investigations of the potential biological interaction of *RAD52* and *FMR1* showed that both genes are highly expressed in the ovary (Supplementary Fig. S4), suggesting a possible shared role in ovarian ageing. Given recent studies highlighting DNA repair genes as putative therapeutic targets for repeat expansion disorders (López Castel *et al.*, 2010; Zhao and Usdin, 2015), future studies should seek to replicate our observation and experimentally evaluate the role of *RAD52* on somatic *FMR1* repeat expansion.

We also examined interactions of the *FMR1* premutation with categorical smoking, alcohol consumption, previous use of oral hormonal contraceptives, number of live births, previous pelvic surgery, and BMI on POI risk, and found no significant interactions with premutation status, despite many of these exposures being independently associated with increased POI risk (Supplementary Fig. S5).

## Discussion

In the largest investigation to date of the impact of *FMR1* CGG repeat length on menopause phenotypes, we confirm a positive association between carrying a premutation at >55 repeat lengths and POI, refining the understanding of premutation thresholds and their predictive value for reproductive ageing phenotypes. In a population-based sample of 92 557 unrelated, European women from UKBB with data on ANM, we found that 1.8% of women with POI carried an *FMR1* premutation, compared to 0.54% in controls. This aligns with estimates smaller-scale population-based studies (Murray *et al.*, 2014), providing robust confirmation in a much larger cohort. We also estimate that only 6.9% of premutation carriers develop POI. The allele length distribution was broadly similar between women with POI and those without. We also estimated that the odds ratio of POI in premutation carriers relative to women with a repeat length of 30 is 3.49, and less than the odds ratio first percentile of the PRS for ANM of 4.71 (Ruth *et al.*, 2021).

Our 6.9% penetrance estimate represents a substantial deviation from prior estimates (12–20%) based on smaller studies, often enriched for family history or clinical ascertainment (Sherman, 2000; Rodriguez-Revenga *et al.*, 2009). This highlights the importance of large, ‘genotype-first’ population-based estimates of *FMR1* repeat length. It also questions the relevance of the term ‘Fragile X-Associated Premature Ovarian Insufficiency’ (FXPOI) given the risk of POI presented by the repeat is similar to that of a common variant polygenic score (Ruth *et al.*, 2021) and we show that the effect of these common variants on POI is the same in premutation carriers than non-carriers. Importantly, we find no evidence of participation bias which would lead women to preferentially join UK Biobank based on their menopausal status. This is in contrast to traits such as BMI, where we previously demonstrated that females with higher genetic susceptibility to obesity are less likely to participate than their male equivalents (Pirastu *et al.*, 2021).

Currently, women with non-iatrogenic POI are offered karyotype and *FMR1* premutation screening, as recommended by the National Genomic Test Directory (R402.1 and R402.2; National Genomic Test Directory Testing Criteria for Rare and Inherited Disease, 2024). While the primary aim of this screening is to identify the underlying cause of POI, its most meaningful utility lies in reproductive counselling. Women with FXPOI can still conceive due to intermittent ovarian function, and identifying a premutation informs the risk of transmitting a fully expanded *FMR1* allele, associated with Fragile X syndrome, to their children (Nolin *et al.*, 2003). This reproductive implication is arguably the strongest rationale for *FMR1* screening in clinical practice given the poor diagnostic performance we demonstrate.

ROC analysis showed poor predictive power of the 55-repeat threshold (AUC = 0.601), lower than the AUC of 0.64 for a menopause polygenic score (Ruth *et al.*, 2021), suggesting low penetrance of the premutation limited value as a standalone biomarker. This weak association underscores the complex relationship between *FMR1* repeat length and ovarian function, likely influenced by factors such as RNA toxicity, polygenic background, and X-inactivation patterns. Similar modifying effects have been observed in other monogenic conditions like hereditary breast cancer (Fahed *et al.*, 2020). As public health priorities shift to genomic screening for risk prediction in healthy individuals (Armstrong, 2021), it is important to critically evaluate *FMR1* screening’s value in this context. These findings suggest that relying solely on repeat length may be insufficient for risk prediction in healthy women. Moreover, the 6.9% POI prevalence in carriers in our cohort, compared to the global estimate of 3.5% for all women, is lower than historical 20:1 risk ratio estimates (Tassanakijpanich *et al.*, 2021). Combining *FMR1* testing with hormonal or clinical risk markers could improve predictive value, but such markers remain undefined. The main clinical benefit of *FMR1* testing may therefore lie in informing reproductive risk rather than guiding POI diagnosis or management.

Many studies have sought to refine the premutation threshold for clinical use. A meta-analysis of 18 studies suggested that within the 55–200 repeat range is associated with elevated risk of POI, but that the intermediate range of 45–54 repeats shows no association with POI (Huang *et al.*, 2019). Using regression models varying the premutation threshold, we identified significant POI odds beginning at 36 CGG repeats, with the most significant association observed at the traditional 55-repeat cutoff (OR = 3.43). These results suggest a continuous risk gradient, and may suggest that women with 36 or more repeats may be more likely to suffer from diminished ovarian reserve. Measurements of anti-Mullerian hormone (AMH) were unavailable in the UK Biobank, but the measurement of AMH levels in women with 36 repeats or more, to determine ovarian reserve, should be a focus of future studies.

However, if a binary threshold is required for clinical decision making, the traditional repeat length of 55 remains the most appropriate based on predictive performance. Higher repeat thresholds, such as 72 repeats, showed even greater effect sizes but involved smaller sample sizes, leading to uncertainties about statistical power and biological interpretation, though this observation aligns with results in other studies that the 70–90 range confers the highest POI risk (Rosario & Anderson, 2020). We also explored the associations between *FMR1* repeat length and other reproductive phenotypes in both sexes, but found no associations. This suggests *FMR1* expansions affect ovarian reserve rather than broader reproductive development.

We modelled the association between *FMR1* repeat length and FMRP protein levels to determine whether the mechanism of action of *FMR1* on POI may be related to protein levels. Previous studies have suggested an inverse association between *FMR1* repeat length and FMRP levels in the premutation range (Kenneson *et al.*, 2001). We found no association, however, between *FMR1* repeat length or *FMR1* premutation status and FMRP levels in either sex. A few alternate theories of action of *FMR1* premutations on FXPOI include an RNA toxic gain-of-function mechanism, in which the expanded CGG repeat tract in *FMR1* mRNA sequesters RNA-binding proteins, leading to cellular stress, mitochondrial dysfunction, and the dysregulation of normal ovarian function (Rosario and Anderson, 2020). Others include a protein-based mechanism, whereby repeat-associated non-AUG translation leads to the formation of FMRpolyG, which is an abnormal polyglycine-related protein (Rosario and Anderson, 2020). Our result of no association between *FMR1* repeat length and FMRP levels supports the hypotheses that RNA gain-of-function toxicity is more likely than FMRP deficiency to be the primary pathogenic mechanism underlying FXPOI.

This study has some limitations. We could not assess AGG interruptions, which stabilize CGG repeat tracts and may influence the risk of expansion due to limitations in ExpansionHunter’s output. However, very few experimental studies of the effect of AGG interruptions on POI have been conducted, and most of those find no relationship between AGG interruptions and POI (Allen *et al.*, 2018; Rodrigues *et al.*, 2024). We could not explore mosaicism or X-inactivation patterns in this analysis, which could modulate functional consequences of premutations. However, in this study, it was not possible to measure mosaicism in the ovary, and tissue-specific mosaicism is known to be a key factor in determining the link between a repeat expansion and the phenotype of interest (Handsaker *et al.*, 2025). Mosaicism measured in the blood would not be relevant for understanding ovary-specific effects of *FMR1* expansions. Currently, the technology to assess X-linked inactivation at the population scale does not exist, though prior evidence shows that X-linked inactivation is not meaningfully associated with POI in the case of *FMR1* (Spath *et al.*, 2010). In addition, we acknowledge that our cases are defined predominantly by self-report rather than clinical ascertainment, however, we have previously demonstrated that genetic analyses of self-reported menopause age recapitulate established ovarian biology from clinical and animal studies (Ruth *et al.*, 2021).

In summary, using UK Biobank data from over 92 000 women, we confirm a robust association between *FMR1* premutation status and POI, but with lower penetrance than has previously been reported. Considering that most women with confirmed POI will have an *FMR1* premutation screen performed, our findings have important implications for clinical practice. We demonstrate that an *FMR1* premutation has only modest predictive power, limiting its diagnostic utility beyond reproductive counselling. Additionally, our findings support a more continuous risk model for *FMR1* premutation status, beginning below the traditional 55-repeat cutoff, and reinforce RNA toxicity, opposed to FMRP deficiency, as the likely pathogenic mechanism. We highlight the important role of the Clinical Geneticist in the multidisciplinary care of women with POI when explaining the nuances of a positive *FMR1* premutation screen result in clinical practice. Considered together, this work challenges the utility of a standalone FXPOI disease category and suggests that *FMR1* repeat expansion, while justified for inclusion as an evidence-based investigation for POI, should not be considered a sole determinant of an individual’s POI diagnosis. Why some women carrying *FMR1* premutations develop POI and others do not would be a useful focus for future research.

## Supplementary Material

deag061_Supplementary_Figure_S1

deag061_Supplementary_Figure_S2

deag061_Supplementary_Figure_S3

deag061_Supplementary_Figure_S4

deag061_Supplementary_Figure_S5

deag061_Supplementary_Table_S1

deag061_Supplementary_Table_S2

deag061_Supplementary_Table_S3

deag061_Supplementary_Table_S4

deag061_Supplementary_Table_S5

deag061_Supplementary_Table_S6

deag061_Supplementary_Table_S7

## Data Availability

The data reported in this paper are available to other investigators by application directly to the UK Biobank. Software code in R for implementing the analyses will be made available upon request.
