# Supplementary material for: Large-scale analysis of FMR1 CGG repeat length and risk of premature ovarian insufficiency in over 92 000 women
Source: Hum Reprod. 2026 Apr 19;41(6):998–1007. doi: 10.1093/humrep/deag061 (PMC13231448; doi:10.1093/humrep/deag061)
Supplement: deag061_Supplementary_Figure_S1 [file deag061_supplementary_figure_s1.pdf]

*Human Reproduction*, 2026, 1–1
https://doi.org/10.1093/humrep/deag061
**Supplementary Data**

human reproduction
OXFORD

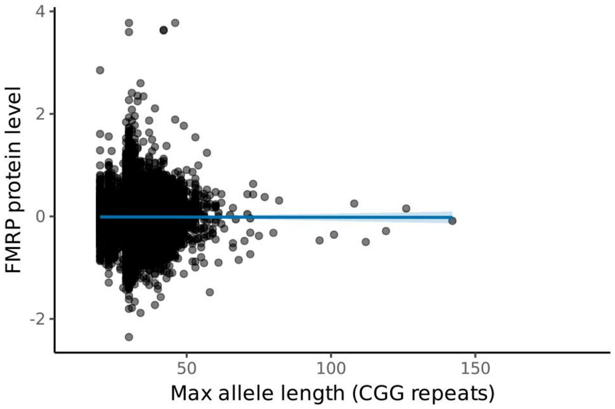


**Supplementary Figure S1. Association between *FMR1* repeat length and FMRP levels in women.** Lack of correlation between *FMR1* repeat length and FMRP protein levels, along with linear regression line depicting an effect estimate of near zero suggests *FMR1* repeat length and FMRP protein levels are not significantly associated with one another. This supports the likelihood that the action of *FMR1* premutations on FXPOI include an RNA toxic gain-of-function mechanism rather than a protein-based mechanism.
