# Supplementary material for: Large-scale analysis of FMR1 CGG repeat length and risk of premature ovarian insufficiency in over 92 000 women
Source: Hum Reprod. 2026 Apr 19;41(6):998–1007. doi: 10.1093/humrep/deag061 (PMC13231448; doi:10.1093/humrep/deag061)
Supplement: deag061_Supplementary_Figure_S2 [file deag061_supplementary_figure_s2.pdf]

Human Reproduction, 2026, 1–1
https://doi.org/10.1093/humrep/deag061
Supplementary Data

human reproduction

OXFORD

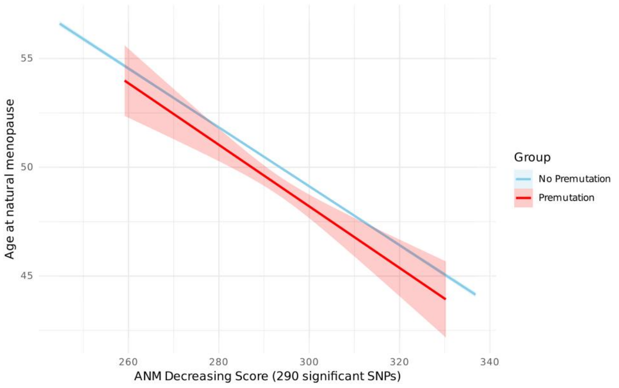

Supplementary Figure S2. Linear regression of the polygenic risk score for age at menopause (derived from 290 genome-wide significant signals for age at menopause) on age at natural menopause in premutation carriers (red) and non-carriers (blue). Shading represents 95% confidence intervals. Overlapping confidence intervals and almost identical directions of effect support the lack of interaction between the 290 signal score for age at menopause with premutation status on age at natural menopause.
