# Supplementary material for: Large-scale analysis of FMR1 CGG repeat length and risk of premature ovarian insufficiency in over 92 000 women
Source: Hum Reprod. 2026 Apr 19;41(6):998–1007. doi: 10.1093/humrep/deag061 (PMC13231448; doi:10.1093/humrep/deag061)
Supplement: deag061_Supplementary_Figure_S3 [file deag061_supplementary_figure_s3.pdf]

Human Reproduction, 2026, 1–1
https://doi.org/10.1093/humrep/deag061
Supplementary Data

human reproduction
OXFORD

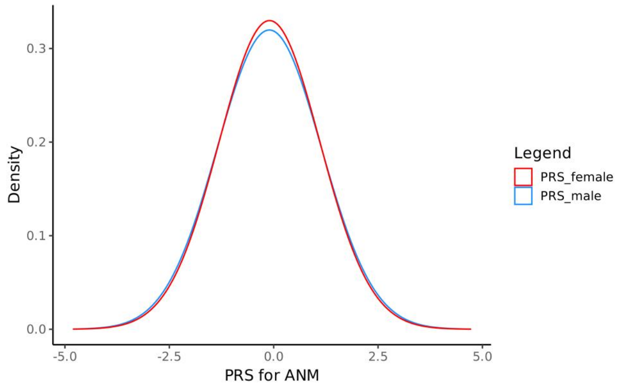

Supplementary Figure S3. Distributions of the polygenic risk score for age at natural menopause in men versus women in the UK Biobank. Distributions of this score are almost identical for men and women, suggesting that there is no participation bias based on menopause age for women in the UK Biobank.
