# Supplementary material for: Large-scale analysis of FMR1 CGG repeat length and risk of premature ovarian insufficiency in over 92 000 women
Source: Hum Reprod. 2026 Apr 19;41(6):998–1007. doi: 10.1093/humrep/deag061 (PMC13231448; doi:10.1093/humrep/deag061)
Supplement: deag061_Supplementary_Figure_S4 [file deag061_supplementary_figure_s4.pdf]

Human Reproduction, 2026, 1–1
https://doi.org/10.1093/humrep/deag061
Supplementary Data
human reproduction
OXFORD

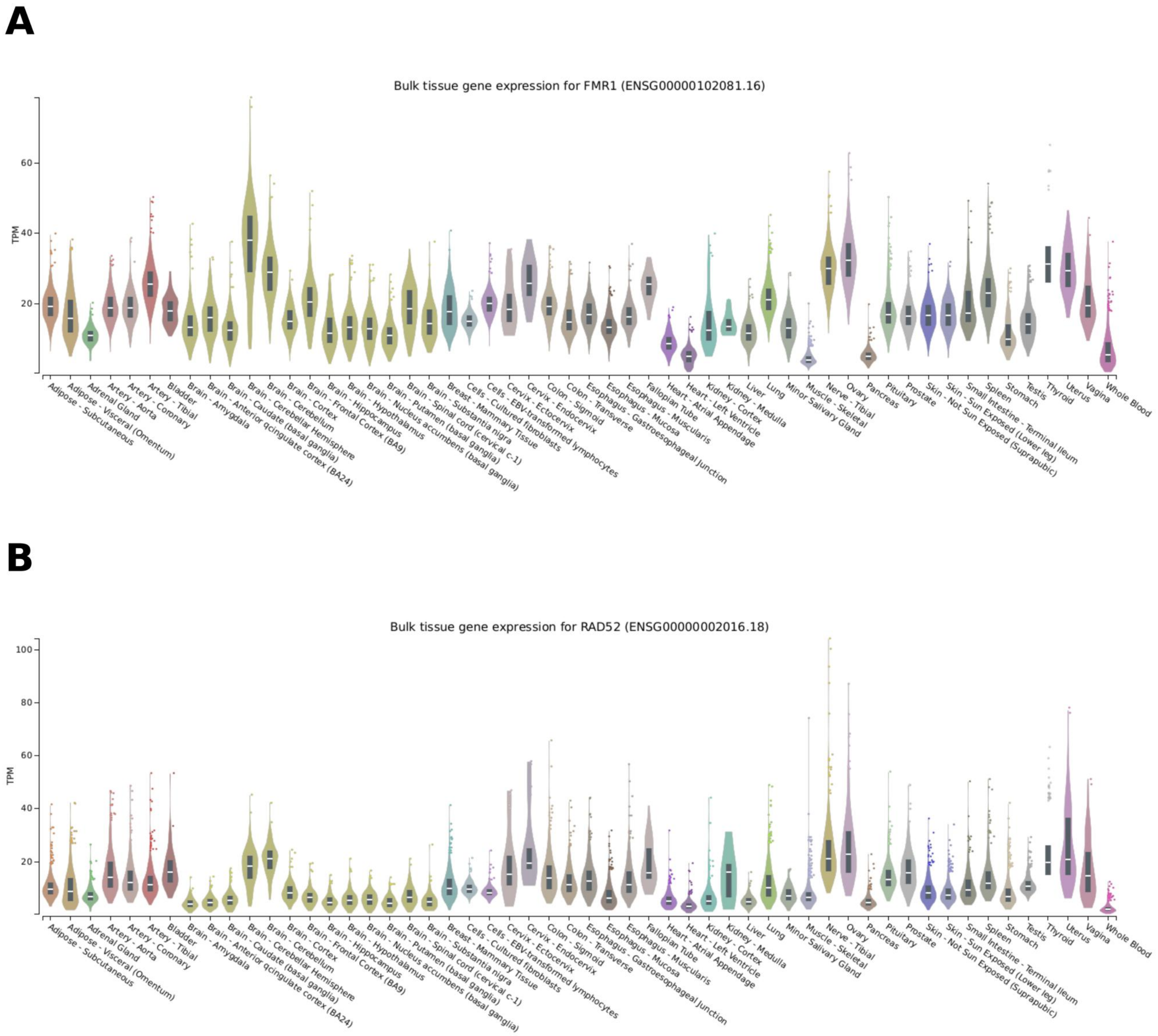

Supplementary Figure S4. **GTEx tissue level expression data for (A) *FMR1* and (B) *RAD52*.** Similarly elevated expression of each gene is shown for the ovarian tissue. This supports a potential for biological interaction between *RAD52* and *FMR1* in line with statistically significant interaction highlighted by interaction models.
