# Supplementary material for: Large-scale analysis of FMR1 CGG repeat length and risk of premature ovarian insufficiency in over 92 000 women
Source: Hum Reprod. 2026 Apr 19;41(6):998–1007. doi: 10.1093/humrep/deag061 (PMC13231448; doi:10.1093/humrep/deag061)
Supplement: deag061_Supplementary_Figure_S5 [file deag061_supplementary_figure_s5.pdf]

Human Reproduction, 2026, 1–1
https://doi.org/10.1093/humrep/deag061
Supplementary Data

human reproduction
OXFORD

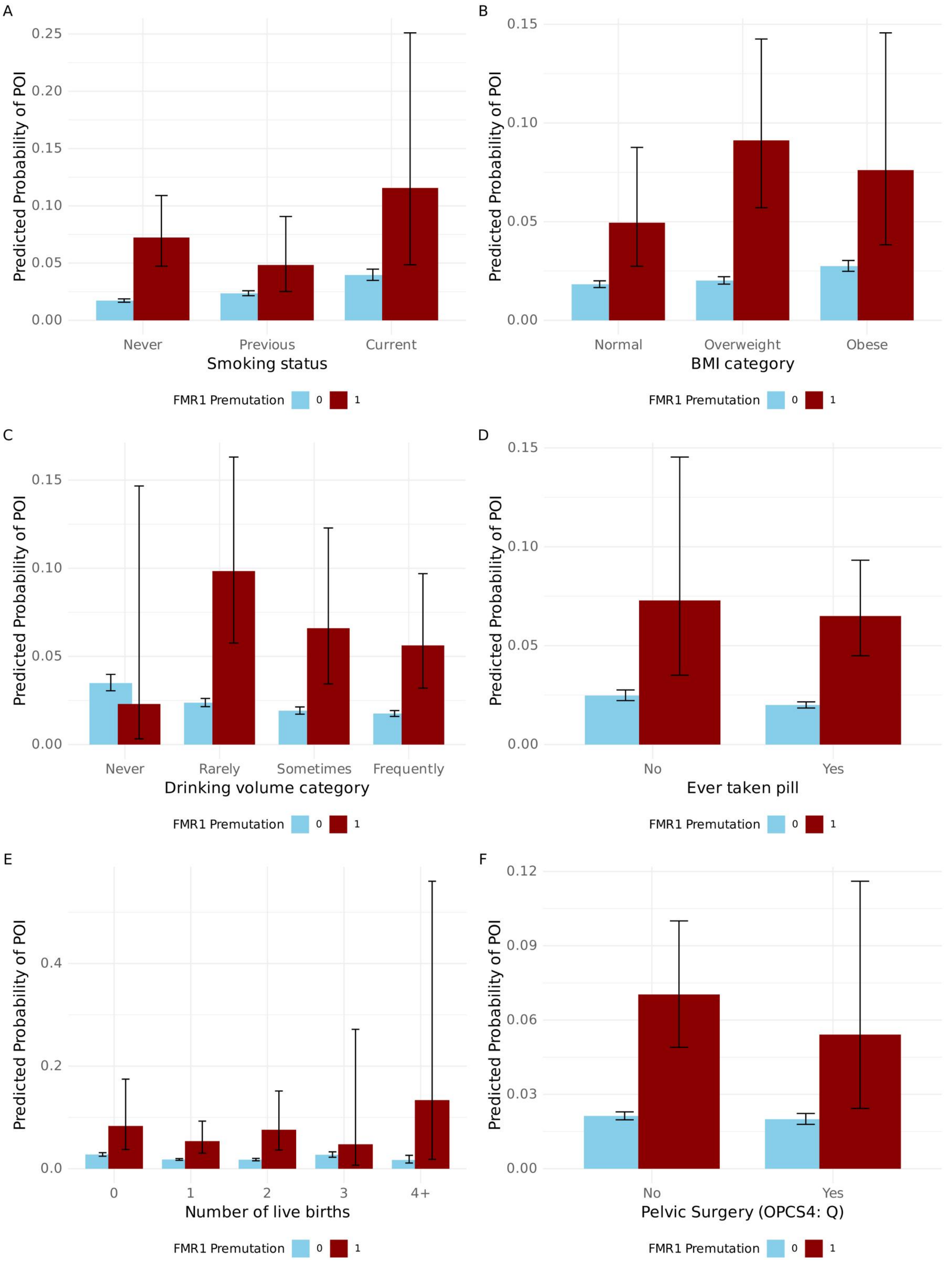

Supplementary Figure S5. **Predicted probability of POI based on environmental exposures (A: smoking status, B: BMI, C: alcohol consumption, D: oral contraceptive use, E: number of live births, and F: pelvic surgery) among premutation carriers and non-carriers in the UK Biobank women.** Bars are coloured by premutation status (red: premutation, blue: no premutation) and 95% confidence intervals are represented by error bars. No clear pattern is observed across different interaction variables, supporting the lack of interaction between premutation status and each of the environmental variables on POI risk.
